# Short-term heat shock perturbation affects populations of *Daphnia magna* and *Eurytemora carolleeae*: a warning to the water thermal pollution

**DOI:** 10.1038/s41598-021-96464-8

**Published:** 2021-08-19

**Authors:** Kacper Nowakowski, Łukasz Sługocki

**Affiliations:** 1 Department of Hydrobiology, Institute of Biology, University of Szczecin, Felczaka 3c, 71-712 Szczecin, Poland; 2 Center of Molecular Biology and Biotechnology, University of Szczecin, Wąska 13, 71-715 Szczecin, Poland

**Keywords:** Freshwater ecology, Ecology, Population dynamics, Zoology, Environmental sciences

## Abstract

Thermal pollution leads to short-term heat shock in aquatic invertebrates; however, the modulation of tolerance and life history of these invertebrates by thermal stress varies among regions, phenology, species, and their acclimation. To assess the effect of thermal shock, we conducted experiments on *Daphnia magna* and *Eurytemora carolleeae* at 25 °C, 30 °C, 35 °C, and 40 °C (in relation to 20 °C) in a different exposure time of the stressor (10, 30, and 60 min). The results showed that short-term heat shock leads to increased mortality and reduced fertility of the studied planktonic crustaceans. *D. magna* was more resistant to thermal shock than *E. carolleeae* according to all variants of exposure based on the calculated LT50 values for 24, 48, and 72 h. Thermal shock decreased the potential of the *Daphnia* population in terms of the total number of births, however, with regard to individual reproductive abilities, the non-lethal heat shock did not reduce the birth rate. Although *Eurytemora* is more sensitive to thermal shock than *Daphnia*, the type of parental care in *Eurytemora* might be more favorable for offspring survival following thermal shock than in *Daphnia*. In *Eurytemora*, despite maternal deaths, a relatively high number of newborns who survived high temperatures were observed. The obtained results can help to understand the ecological processes occurring due to anthropogenic thermal pollution.

## Introduction

Anthropogenic stressors pose a serious threat to microinvertebrate fauna in aquatic systems^1,2^. The recognition of disturbance thresholds is essential for the preservation of biodiversity and the stability of communities^3^. An increased temperature that could lead to thermal pollution is one of the most important stressor^4^. Drastic thermal perturbations in aquatic ecosystems are usually related to cooling systems of installations, which discharge heated waters into rivers, lakes, estuaries, or seas^5,6^. In the era of anthropogenically induced climate change, the thermal stress associated with installations using water from natural surface waters will continue to increase^7^. Lowering of water levels in lakes and rivers due to anthropogenic activity in catchments and global warming rapidly increases water temperature^8^. Thermal pollution can lead to a sudden short-term heat shock that affects aquatic organisms. This applies to both vertebrates and small organisms that are carried through the river current^4^. Smaller organisms such as planktonic microinvertebrates or fish larvae cannot actively avoid thermal stress because they are unable to move effectively from the polluted zone. Therefore, in places where heated waters come into contact with natural waters, unnatural conditions emerge, that may adversely affect the communities of aquatic organisms downstream.

Locally warmed zones formed by cooling waters differ greatly from thermal conditions in natural waters^9^. The difference between temperature of intake and discharge water is usually between 4 and 15 °C^9,10^. The most common temperature difference between the intake water and the discharge water in the United States is between 8 and 12 °C; however, there are also power plants that discharge water at 15 °C higher temperature than that of intake water^10^. The temperature of discharge water also differs greatly depending on the installation and climate. For example, reports on nuclear power plants located in northern Taiwan revealed that the temperature of the heated waters can reach 42 °C at the discharge point. The average temperature of heated water discharged from a power plant in the United States was 37 °C^10^. As the water masses move away from the heat source, the water gradually cools down; hence, the thermal stress caused by heat shock may affect planktonic organisms for a short term.

Studies on the influence of heated waters on aquatic ecosystems have usually focused on the species composition of communities (including invasive species), abundance, size of individuals and other features of communities associated with heated waters^5,11,12^. Less attention has been given to the modulation of life histories of certain species affected by short-term heat shock. Different species and even different genotypes among the same species may show different patterns of resistance to thermal stress^6,13^ therefore, the study of the life history of certain species can help to understand the changes occurring in polluted ecosystems. Planktonic crustaceans are an important element of aquatic ecosystems. The two main groups of planktonic crustaceans in freshwater are Cladocera and Copepoda. Representatives of these taxa can respond differently to thermal changes in the environment. Short-term heat shock studies for investigating population characteristics have been conducted on *Daphnia hyalina* but the studies considered only a short period after the heat shock^14^. *D. magna* was used as a model species in thermal pollution studies on a molecular level^13^ but less is known about population response to the stressor. Calanoid copepods as *Acartia, Calanus, Labidocera,* and *Pseudodiaptomus* were used in acute thermal pollution studies^6^ but still less is known about genus *Eurytemora*. Experimental studies on the influence of heat shock on representatives of these taxa are important to understand how a short-term thermal shock affects the formation of heated water communities. Therefore, for the present experiment, model species of crustaceans were chosen, namely *Dapnia magna* Straus 1820 and *Eurytemora carolleeae* Alekseev & Souissi 2011 (*Eurytemora affinis* species complex).

While *Daphnia* can reproduce parthenogenetically, *Eurytemora* is exclusively dioecious. *Daphnia* parental care is different from that of *Eurytemora*. The former protects the eggs in the brood chamber inside the body shell, while the latter protects the eggs in the egg sacs outside the body. Newborn *Daphnia* rely on the protection of the mother chamber from few hours of birth to up to few days, whereas *Eurytemora* could drop the egg sacs and allow the nauplii to be born independently. Hence, if mother parent of *Daphnia* dies (in unfortunate events), it prevents the escape of juveniles from the chamber. In accordance with the aims of present research, the following research hypotheses were formulated:

1. Short-term heat shock leads to increased mortality and reduced fertility in *D. magna* and *E. carolleeae*.
2. Longer exposure to heat shock has negative effects on reproduction and survival of *D. magna* and *E. carolleeae*.
3. The parental care of *Eurytemora* favors the survival and hatching of active eggs after the heat shock as compared to that of *Daphnia*.

## Results

### Survival due to thermal shock

**Figure 1:**
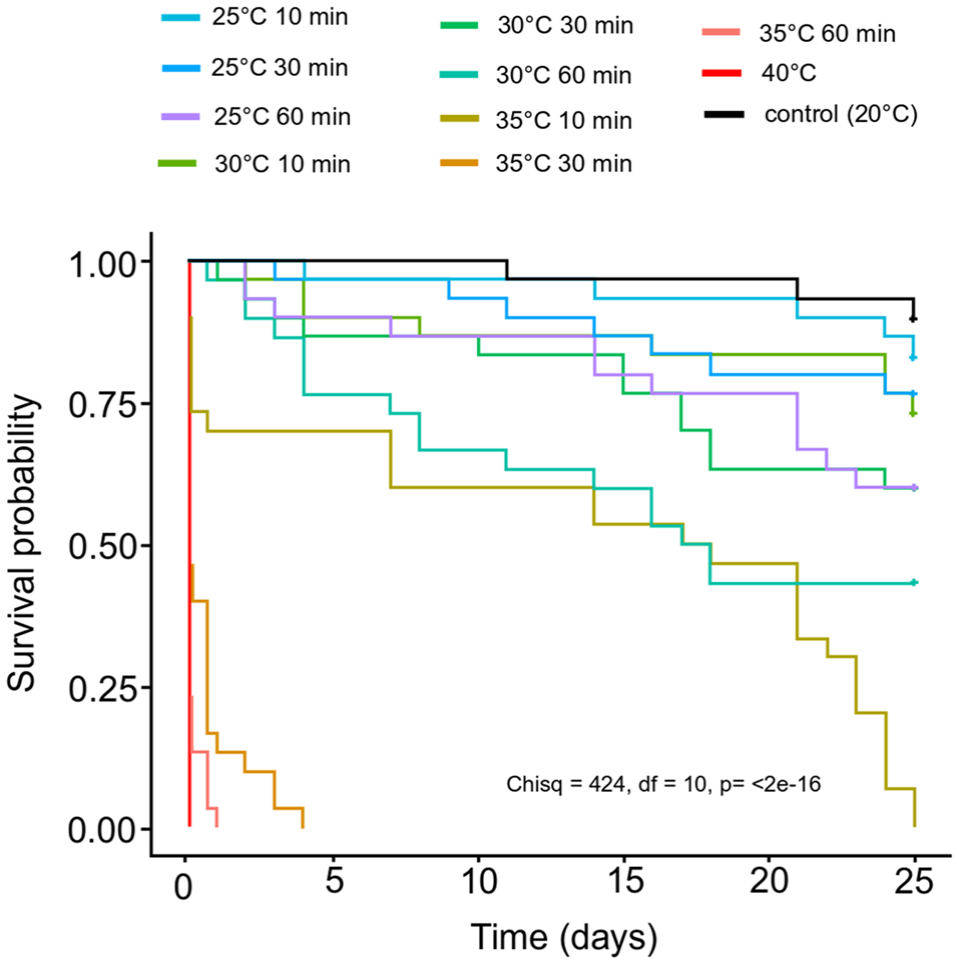
Kaplan–Meier survival curves for *Daphnia magna* exposed to temperature shock for different time periods. Log-rang test with *p* < 0.05.

The survival of *D. magna* depended on the temperature of the heat shock and the exposure time (Fig. 1). The higher the heat shock value, the higher was the mortality of *D. magna* at all the selected exposure times. The survival was lower with prolonged exposure to elevated temperatures, and this trend was clearly visible for heat shock at 25 °C, 30 °C and 35 °C. 25 days after the start of the experiment 90% of specimens survived in the control conditions. At 25 °C heat shock the survival of *Daphnia* on 25 day of experiment ranged from 60% (60 min exposure) to 83% (10 min exposure). At 30 °C heat shock the survival of *Daphnia* on 25 day of experiment ranged from 43% (60 min exposure) to 60% (10 min exposure). At 35 °C and 40 °C heat shock all specimens died until the 25-th day of the experiment.

**Figure 2:**
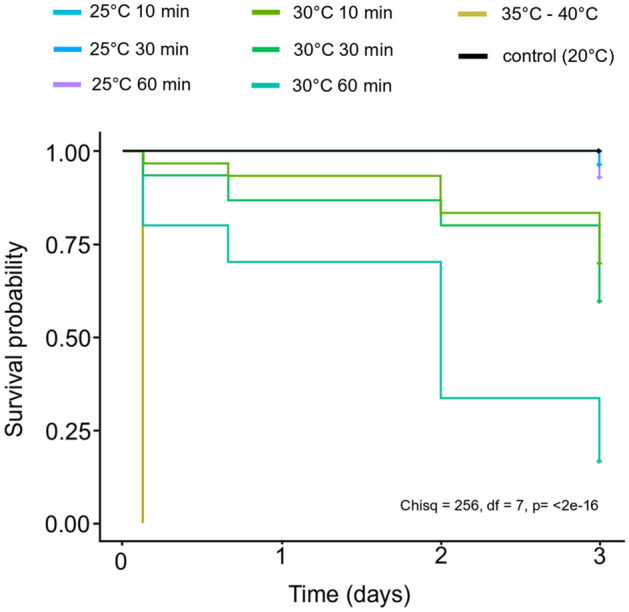
Kaplan–Meier survival curves for *Eurytemora carolleeae* exposed to temperature shock for different time periods. Log-rang test with *p* < 0.05.

The survival in *E. carolleeae* was also dependent on the temperature of heat shock and the time of exposure (Fig. 2). The higher the exposure temperature, the higher was the mortality for *E. carolleeae*. At temperatures of 35 °C and 40 °C, all specimens died in the first hours from the beginning of the experiment. Survival was lower for prolonged exposure to elevated temperature. 3 days after the start of the experiment all specimens survived in the control conditions. At 25 °C heat shock the survival of *Eurytemora* on the 3-rd day of the experiment ranged from 97% (60 min exposure) to 100% (10 min exposure). At 30 °C heat shock the survival of *Eurytemora* on the 3-rd day of the experiment ranged from 20% (60 min exposure) to 70% (10 min exposure).

### LT50 at 24, 48, and 72 h

Thermal tolerance of the experimental microcrustaceans was significantly influenced by the exposure time. LT50 of *D. magna* and *E. carolleeae* decreased with the increase in the exposure time at 24, 48, and 72 h after exposure (see Supplementary Table S1 online). The highest LT50 for *D. magna* was at 10-min exposure after 24 h (38.5 °C), whereas the lowest at 60-min exposure after 72 h (31.8 °C). Similarly, for *E. carolleeae* the highest LT50 was at 10-min exposure after 24 h (33.5 °C), whereas the lowest at 60-min exposure after 72 h (28.7 °C).

### Birth rate of D. magna

The birth rate was not consistent with the total birth of *Daphnia*. The highest birth rate (the mean number of neonates per mother) was observed in 35 °C thermal shock variant for the exposure time of 10 min but the high maternal deaths in this variant unable to include these results in statistical analysis. The lowest birth rate was noted for 30 °C thermal shock variant for 30-min exposure time. Thermal shock had not a significant effect on the birth rate (one-way ANOVA F_5,84_ = 1.67; *p* = 0.1361).

### Egg sack release of E. carolleeae following heat shock

**Figure 3:**
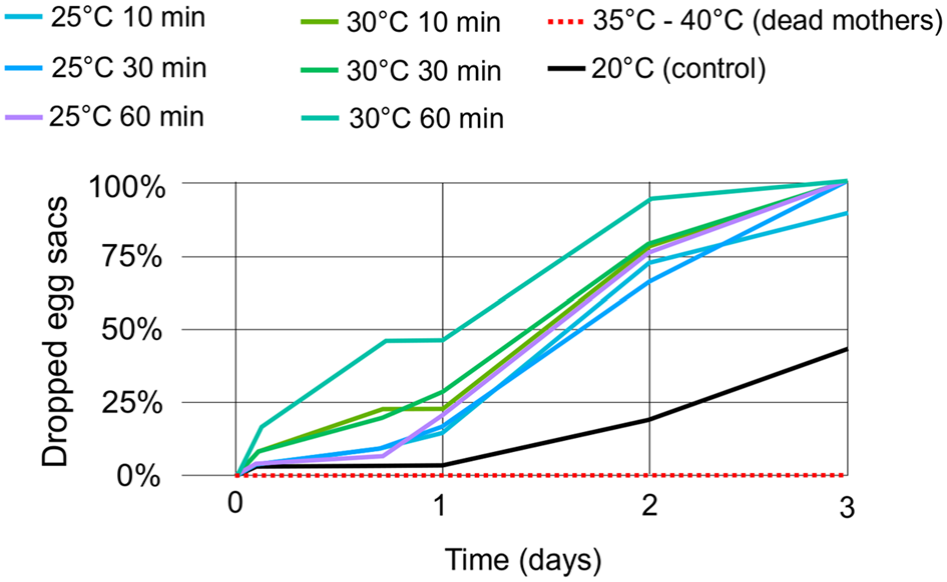
Dropped eggs sacs by *Eurytemora carolleeae* exposed to temperature shock for different time periods.

Generally, the highest the thermal shock and the exposure time, the faster was the release of egg sacs (Fig. 3). At 35 °C and 40 °C, for all exposure time variants, no specimens could release their eggs because all individuals had already died within the first hour of the experiment. In all variant of exposure time at 25 °C and 30 °C, all egg sacs were dropped three days after the start of the experiment, while 43% of the individuals in the control sample released their egg sacs. At 25 °C, the number of released egg sacs was similar at all exposure times. In the 60-min exposure time at 30 °C, the number of dropped egg sacs was 13% after 3 h, whereas it was 0% at 25 °C. After 16 h from the start of the experiment, the number of egg sacs dropped was 47% at 30 °C, but only 7% at 25 °C.

### Total birth of D. magna and E. carolleeae offspring

**Figure 4:**
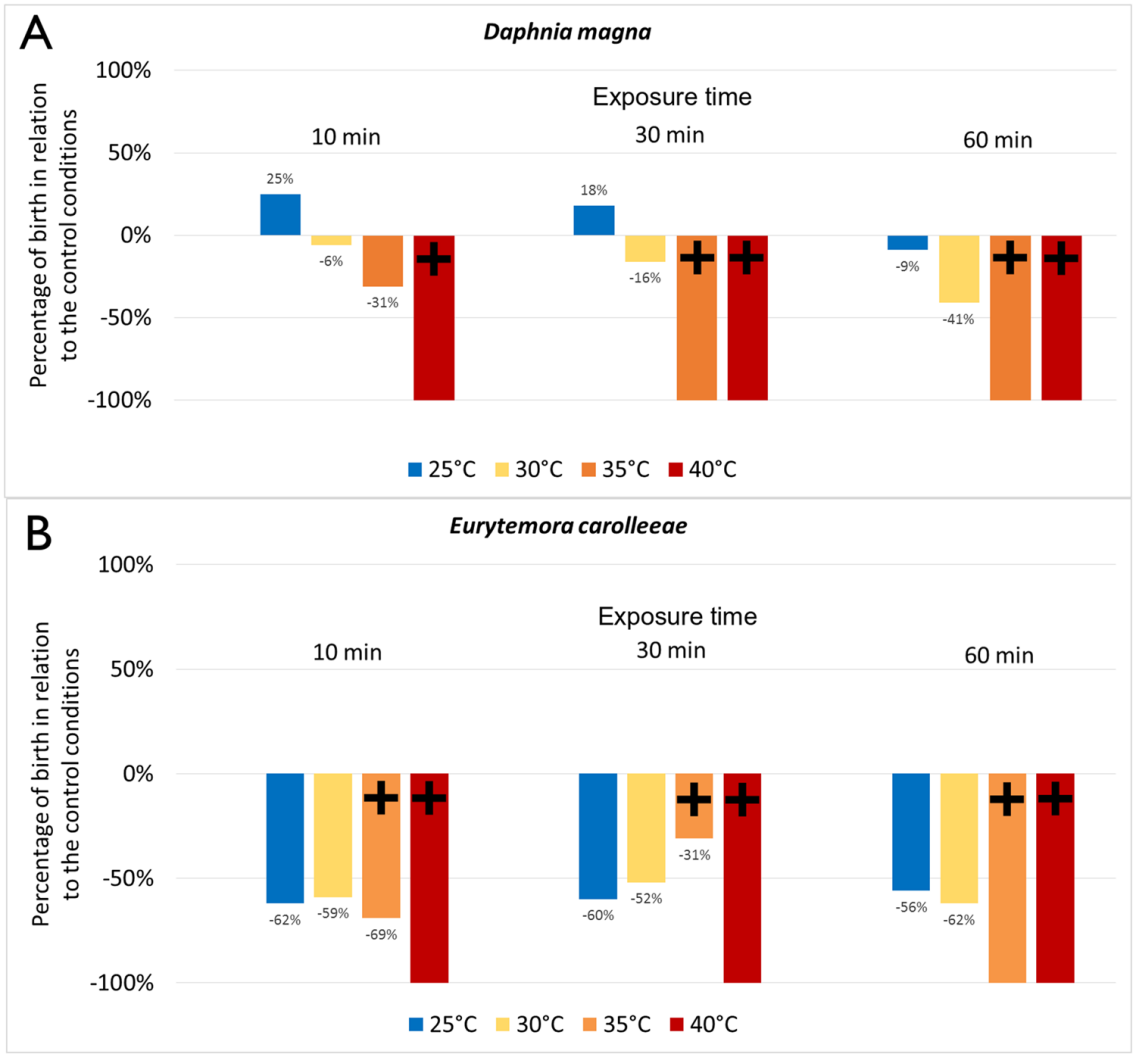
Percentage of changes in the number of births of *Daphnia magna* (**A**) and *Eurytemora carolleeae* (**B**) relative to the control conditions. Black crosses indicate variants of the experiment where all mothers died before the first neonates were born.

The total number of offspring of *D. magna* and *E. carolleeae* depended on the variants of heat shock and exposure time (Fig. 4). The results are presented according to the ratio of the total born specimens in variants of the experiment to the total number of neonates born under control conditions. In all variants of exposure time at 40 °C, no neonates were observed in both *D. magna* and *E. carolleeae*. This phenomenon was also observed for 35 °C variant with 60-min exposure time. At 35 °C and 30 min of exposure time, there were zero newborns in *D. magna,* but a high fertility was observed in *E. carolleeae* (despite the deaths of adults). Thermal shock variants 25 °C and 30 °C in all exposure times decreased the total number of offspring in *E. carolleeae* on average by 50%. We observed different patterns in *D. magna*. Low thermal shock (25 °C) and a short exposure time led to an increase in the number of offspring of *Daphnia*. A short-term thermal shock of 30 °C had a minor effect on the total number of newborns of *Daphnia,* but the total number of offspring decreased as the exposure time increased.

## Discussion

Thermal shock has a pronounced effect on the survival of microcrustaceans, which transfer into the lower potential for population growth. Even a small difference in the time of heat shock exposure leads to changes in the characteristics of the crustaceans populations. Survival of the next generation after exposure of mothers to heat shock is more probable in species that hatch independently on the maternal organism.

According to "Law of Tolerance" formulated by Shelford^15^, species show varying degrees of tolerance to environmental factors, and if the values of a certain factor exceed the tolerance limit, the organism dies. It is presumed that eurythermic species are more resistant to withstand a wide range of thermal conditions. For example, *Daphnia* species growing in temperate climates are less tolerant to higher temperatures than those growing in tropical climates^16^. Some studies on crustaceans have confirmed this pattern^6^; however, other studies have shown contrasting results^17^. Therefore, understanding the tolerance of aquatic species can contribute to a better understanding of how natural fluctuations and human activities affect aquatic communities in certain ecosystems.

We observed that the higher the thermal stress and the exposure time, the higher was the mortality rate in *D. magna* individuals. *D. magna* was more resistant to thermal stress than *D. hyalina* from previous studies^14^. *D. magna* mortality 24 h after one hour of exposure at 30 °C was 3%, but in the case of *D. hyaline* it was 22%. Hence, the thermal tolerance is expected to depend on the habitat and geographic range of the species. In general, the thermal shock led to a decline in the number of *D. magna* individuals (an exception was a low thermal shock, which is described below), but the thermal shock had no effect on the rate of reproduction (in the non-lethal temperature range). This implies that those individuals who survived the thermal stress had similar or higher reproductive abilities than those exposed to mild thermal shock. This phenomenon can be explained by the fact that individuals in worse condition (low fitness) did not survive the thermal stress. The survived specimens were in a better condition, and their high reproductive capacity was observed. Throughout the experiment, the individuals did not suffer from food deficits; therefore, resources do not appear to have played a role in shaping the birth rate in the studied *Daphnia*, as would be expected in conditions with food deficits^18^. Population density may affect individual sizes (less crowded *Daphnia* grow larger in size), which may translate into greater reproduction in *Daphnia*^19^. Hence, the greater birth rate in *Daphnia* in higher thermal shock condition could result from the crowding effects in variants of the experiment where the death rate was very low. However, the crowding effect also seems to play a minor role as the population densities in the experimental containers were low, and in previous studies, similar abundances of *Daphnia* did not demonstrate the crowding effect^20^.

The greater number of births at 25 °C (5 °C shock) than that under the control conditions is most likely because *Daphnia*, which when subjected to a short-term shock, shows increased metabolism that causes the body to respond to rapid growth, at the expense of smaller body size^21,22^. This mechanism allows the body to reproduce as quickly as possible. Reaching reproductive capacity more quickly enables the birth of as many young as possible before death, which is premature in the case of heat shock, thus increasing the probability of survival of the species. The effect of accelerated growth also has a certain limit, after which the growth and development are rapidly inhibited; this finding was also previously demonstrated^23^ and confirmed in the present study.

Heat shock studies have not been conducted very frequently on the copepods^24^, and most of the studies concern the physiological response of the organism as a result of heat shock^25^. Short-term heat shock studies for investigating population characteristics have been conducted for example on calanoid copepods: *Acartia pacifica*, *A. spinicauda, Calanus sinicus, Labidocera euchaeta,* and *Pseudodiaptomus marinus*^6^. To date, no detailed studies have been conducted on the genus *Eurytemora* with regard to the effect of short-term heat shock on mortality and reproduction in a wide range of temperatures. The effect of gradual changes in temperature on *Eurytemora* has been previously studied. Previous studies showed that a gradual increase in temperature by an average of 1–2 °C every 5 min allows the proper functioning of the population of *Eurytemora affinis* and high survival even at 30 °C^26^. However, our results showed that a temperature of 30 °C (10 °C shock) and 60 min of exposure significantly contributed to the increase in mortality (80%) of *E. carolleeae*. Relatively small thermal stress led to the death of *E. carolleeae* as compared to *D. magna*. However, the exposure duration and the temperature did not translate linearly into a decrease in the reproduction of *E. carolleeae*. Despite the death of adult *Eurytemora* specimens due to thermal shock, without having time to release their eggs sacs, it did not lead to the death of the eggs; this resulted in relatively high percentage of births as compared to that under the control conditions. This finding demonstrates the advantage of Copepoda (and especially Calanoida) over other invertebrates in surviving under adverse conditions. Parental care such as that in Calanoida, i.e., keeping the eggs in the egg sacs outside the body, is safer for the offspring than in the case of Cladocera, where the eggs are hidden in the breeding chamber, and if the mother dies, it also leads to the death of the offspring. Hence, the survival of Copepod eggs despite maternal deaths has been repeatedly observed. For example, in fish-eaten copepods, the eggs that pass through the digestive system of the fish have been shown to be still active after escaping back into the water^27^. The authors also observed higher survival rate for Calanoida than for Cyclopoida (because calanoids have ability to produce resting eggs). Cladocera produces diapause eggs (ephippia) under certain phonological cycles or prolonged stress in the environment, but many Calanoid species regularly produce resting eggs^28^. Therefore, the difference in parental care between Cladocera and Copepoda could also play role in population growth in ecosystems threatened with thermal pollution or other significant stressors.

The higher tolerance to thermal stress (LT50) of *D. magna* than that of *E. carolleeae* may be because in our experiment, we tested *Daphnia* and *Eurytemora* individuals that were not of identical age. Studies showed that effects of higher temperature on younger *Daphnia* individuals have less impact on survival and proper functioning, unlike older individuals^21^. Other studies on mortality of *Eurytemora americana* in cooling systems showed higher mortality of juveniles and thus lower resistance of juvenile to thermal stress^29^. Therefore, the results obtained in the present study should not be directly transposed to other developmental stages of microinvertebrates. It also should not be generalized to certain genus or species, because different species may be characterized by different resistance patterns^3,12^. Moreover, the tolerance of the species to heat shock could be related to certain genotypes, which was demonstrated based on *Daphnia pulicaria* studies^13^ and acclimation to certain thermal conditions^6^.

Permanently elevated temperature in aquatic systems could lead to adverse changes in zooplankton communities^7^. Thermal pollution in rivers due to discharge of cooling water seems to be crucial in the formation of invertebrate communities not only below the outflow of heated water from the power plant but also above it. During shaping of the communities below the outflow of heated waters, the species less resistant to thermal shock might disappear, simultaneously creating conditions for the survival of eurythermic, tropical, and sometimes invasive species. It seems that thermal pollution in the river may also indirectly affect communities above the discharges, as a result of hindering upstream migration of species less resistant to thermal pollution. Long-term survey of the zooplankton communities in an aquatic system near a power plant in North America showed higher sensitivity to plant passage of calanoid copepods (*Eurytemora affinis, Limnocalanus macrurus*, and *Diaptomus* spp) than cladocerans (*Daphnia* spp, and *Eubosmina coregoni*)^5^. This is in accordance with our experimental results. *E. carolleeae* was more sensitive to heat shock than *D. magna*. *D. magna* is found in various parts of the world, which implies that the species should have a higher range of adaptation to various thermal conditions than *E. carolleeae* that inhabit only the Northern Hemisphere. Increased mortality probably results from physiological stress, which is the response of an organism to an induced heat shock that causes changes in the body.

These changes could result from direct effects on macromolecules (protein damage)^30^, tissue hypoxia (mismatch between oxygen supply and demand)^31^, formation of reactive oxygen species^32^, impairment of proteins in the endoplasmic reticulum^33^, and reduction of integrity of heat-shock proteins (HSPs)^34^. The change in HSP levels is one of the well-known responses of the body after induction of heat shock^35^. Changes in the level of these proteins as a response to heat shock in *D. magna* were previously reported^36^. These proteins protect the cellular structure against thermal stress and repair proteins damaged by thermal shock^35^. If the body does not maintain with the production of stress proteins or is unable to produce adequate amount of these proteins, cell damage occurs, that translates into a malfunction of the body^35–37^. The reason why *Daphnia* shows more endurance is because this species has a better defense mechanism against physiological stress, which possibly enables it to rapidly produce stress proteins to protect the body against cell damage and allow repair of damaged proteins. However, in *Eurytemora,* which exist in cooler waters, its body most likely does not produce adequate stress proteins to prevent thermal shock-induced damage to the body or other physiological changes, leading to a higher death rate of this species as compared to that of *Daphnia*. Previous studies on physiological responses to short-term heat stress in *Eurytemora affinis* demonstrated that the levels of grp78 and hsp90 transcripts increased after heat-shock from 19 to 28 °C, and the peak level was recorded at 1.5 h after heat shock induction^25^. The authors also revealed that HSP70 increased with an increase in acute temperature exposure and that these proteins were detectable for 30 h after heat shock.

The results of the present study could be useful to formulate regulations regarding the temperature limits in the power plant cooling systems in the warm season. The obtained data would also be useful in planning electrical industries and to conduct environmental assessments of new power plants for reducing their harmful effects on aquatic ecosystems. Thermal stress may have different effects on individual taxa. Further studies on other taxa are needed for a better understanding of the thermal pollution on the natural microcrustaceans communities. We speculate that further research will show common features at taxonomic levels and will depend on the distribution range of individual species. Still the open question is how short thermal perturbations work with other important stressors in thermally polluted habitats as deficits of oxygen, cyanotoxins, or other anthropogenic perturbations. The most probable effect of these stressors is synergy what could endanger the functioning thermally polluted habitats.

## Methods

Four variants of thermal shock, namely 25 °C, 30 °C, 35 °C and 40 °C in relation to the reference conditions (20 °C) were selected to study the effect of short-term heat shock on planktonic crustaceans *Daphnia magna* Straus, 1820 and *Eurytemora carolleeae* Alekseev and Souissi, 2011. *D. magna* was obtained from cultures established using specimens collected (September 19, 2014) from a pond located in north Poland (53° 44′ 47.6″ N, 17° 31′ 51.6″ E). *E. carolleeae* was obtained (September 04, 2020) from the Oder river, Poland (53° 25′ 47.2″ N, 14° 34′ 21.4″ E). *E. carolleeae* was previously identified as an invasive and non-native species in European estuaries and fresh water basins^38,39^. *D. magna* is an eurythermic species, while *E. carolleeae* is found only in the Northern Hemisphere; hence, *Daphnia* has a wider climatic range and is therefore expected to have a larger tolerance to thermal stress.

For *Daphnia magna*, because the experiment lasted for 25 days, the individuals were fed regularly (1 µg of powder *Chlorella* sp. per container every alternate day). *Eurytemora* individuals were not additionally fed because the experiment on this taxon lasted for 3 days. During the experiment, *Daphnia* individuals were kept under the same water conditions as used in the culture (mineral water Lewiatan; conductivity 643 ± 24 µS cm^−1^, oxygen concentration 7.54 ± 0.63 mg L^−1^, pH 7.74 ± 0.11, temperature 20 ± 0.8 °C). In *Eurytemora* experiment, we used filtered water (mesh size: 100 µm) from the same place from where the specimens were obtained (Oder river; conductivity 649 µS cm^−1^, oxygen concentration 6.83 mg L^−1^, pH 7.51, temperature 17.5 °C). Newborns of *Eurytemora* were counted from the completion of the experiment for the next 5 days. Newborns of *Daphnia* were counted (and transferred to another container) from the beginning of the experiment until 25th day of the experiment (every day excluding weekends). Here, water losses were replenished with mineral water used for the cultivation of *Daphnia*. Before classifying the specimen as dead, its vital functions were checked twice under a microscope (during counting and 24 h after the first check to confirm the death).

The water was heated in a paraffin bath (Microm, SB 80) with an accuracy of 0.1 °C. The vessels with water were placed in the bath, and after attaining a certain temperature, *D. magna* and *E. carolleeae* individuals were introduced into the heated container for a defined period of time. To monitor the accuracy of the temperature values, we used a multifunctional field laboratory device (Elmetron CX-401). The oxygen concentration in the heated containers was also controlled, but no significant decrease in oxygenation was observed. For all temperature variants, three exposure times were established: 10, 30, and 60 min. Thermal shock conducted on daphnids and copepods has shown that the acclimation temperature has a large effect on the tolerance of individuals of a given species^6,25,40^. Therefore, in the experiment, individuals under similar thermal conditions were used by carrying out their acclimation. Twenty four hours before the experiment, all specimens were acclimated to the laboratory condition. For each variant and exposure time, 30 individuals were gently selected and placed in three separate 120 ml containers (10 individuals in each container). For the experiment, we used only females: 360 *Daphnia* specimens and 360 *Eurytemora* specimens. In the experiment on *Daphnia*, juvenile of about 1.1–1.4 mm in size were used (5–7 days old). Specimens of this size were selected because they had not yet produced eggs, which enabled the birth rate to be controlled from the first litter. We excluded newborns from the experiment because their mortality could be forced by stochastic events, as noted in our preliminary experiments. For dioecious *Eurytemora*, we examined only adult females with egg sacs. This was because determining the sex of juvenile copepod (without stressing the animal) is difficult. After thermal shock was induced, crustaceans were placed under stable laboratory conditions at approximately 20 °C. To provide the crustaceans with optimal conditions during the experiment, all individuals were placed under the same lighting conditions (LED light, white color 6 W; 16 h light/8 h dark).

To plot the differences in survivorship among the variants of the experiment a Kaplan–Meier analysis was used. Log-rank test was performed to test differences between variants of the experiment (R version 4.0.5). Temperature leading to the death of 50% of *Daphnia* and *Eurytemora* individuals (LT50) was calculated for different exposure durations by using logarithmic regression. The birth rate of *D. magna* was calculated as the mean number of neonates per mother on each day of the experiment. One-way ANOVA was used to find significant differences between the birth rate of the control group and that of the experimental group subjected to variants of thermal shock and exposure time (Statistica 13.1, Statsoft).

## Supplementary Information

Supplementary Information.

## Data Availability

The datasets generated during and/or analyzed during the current study are available from the corresponding author on reasonable request.
