# Supplementary material for: Short-term heat shock perturbation affects populations of Daphnia magna and Eurytemora carolleeae: a warning to the water thermal pollution
Source: Sci Rep. 2021 Aug 19;11:16909. doi: 10.1038/s41598-021-96464-8 (PMC8377013; doi:10.1038/s41598-021-96464-8)
Supplement: Supplementary file 1 — Supplementary Information. [file 41598_2021_96464_MOESM1_ESM.docx]

Short-term heat shock perturbation affects populations of *Daphnia magna* and *Eurytemora carolleeae*: a warning to the water thermal pollution

Kacper Nowakowski^1^, and Łukasz Sługocki^1,2,^*

^1^Department of Hydrobiology, Institute of Biology, University of Szczecin, Felczaka 3c, 71-712 Szczecin, Poland

^2^Center of Molecular Biology and Biotechnology, University of Szczecin, Wąska 13, 71-715 Szczecin, Poland

*Corresponding author: [lukasz.slugocki@usz.edu.pl](mailto:lukasz.slugocki@usz.edu.pl)

Key words: Thermal pollution, Cooling systems, Thermal tolerance, Acute thermal stress, Zooplankton, Microinvertebrates, *Daphnia*, Calanoida, Crustacea

**Supplementary Information**

**Table S1**. LT50 calculations based on the lethal rate with different exposure durations. The numbers in bold indicate absolute temperature leading to the death of 50% of the population, and the values in parentheses show the difference value between heat shock and control conditions.

|  |  |  | Exposure 10 min | Exposure 30 min | Exposure 60 min |
| --- | --- | --- | --- | --- | --- |
| *Daphnia magna* | LT50 24h | Temp. (°C) | **38.5 (18.5)** | **33.7 (13.7)** | **33.5 (13.5)** |
|  |  | Formula | 30+5*log10(x) | 24.7406+5.2948*log10(x) | 25+5*log10(x) |
|  |  | Regression value | r = 0.91; p = 0.0007 | r = 0.87; p = 0.0021 | r = 0.88; p = 0.0018 |
|  | LT50 48h | Temp. (°C) | **38.1 (18.1)** | **33.7 (13.7)** | **32.4 (12.4)** |
|  |  | Formula | 27.5+6.25*log10(x) | 24.7858+5.2271*log10(x) | 19.8987+7.3534*log10(x) |
|  |  | Regression value | r = 0.89; p = 0.0012 | r = 0.89; p = 0.0013 | r = 0.82; p = 0.0012 |
|  | LT50 72h | Temp. (°C) | **38.1 (18.1)** | **32.9 (12.9)** | **31.8 (11.8)** |
|  |  | Formula | 27.5+6.25*log10(x) | 19.9942+7.567*log10(x) | 20.8995+6.4411*log10(x) |
|  |  | Regression value | r = 0.89; p = 0.0012 | r = 0.82; p = 0.0011 | r = 0.83; p = 0.0009 |
| *Eurytemora carolleeae* | LT50 24h | Temp. (°C) | **33.5 (13.5)** | **33.2 (13.2)** | **32.9 (12.9)** |
|  |  | Formula | 25+5*log10(x) | 23.714+5.5933*log10(x) | 22.8616+5.9143*log10(x) |
|  |  | Regression value | r = 0.89; p = 0.0012 | r = 0.90; p = 0.0009 | r = 0.94; p = 0.0002 |
|  | LT50 48h | Temp. (°C) | **32.9 (12.9)** | **32.9 (12.9)** | **28.8 (8.8)** |
|  |  | Formula | 22.8616+5.9143*log10(x) | 22.8616+5.9143*log10(x) | - 4.1407+19.4171*log10(x) |
|  |  | Regression value | r = 0.94; p = 0.0002 | r = 0.94; p = 0.0002 | r = 0.99; p = 0.00000 |
|  | LT50 72h | Temp. (°C) | **32.4 (12.4)** | **31.6 (11.6)** | **28.7 (8.7)** |
|  |  | Formula | 20.0699+7.2401*log10(x) | 14.5973+10.0032*log10(x) | 19.8149+5.2564*log10(x) |
|  |  | Regression value | r = 0.96; p = 0.00005 | r = 0.98; p = 0.00000 | r = 0.87; p = 0.0025 |
